# Qualitative and Quantitative Characterization of Microplastics Released from Infant PET Outerwear Using ICP-AES and FTIR

**DOI:** 10.3390/molecules31142467

**Published:** 2026-07-15

**Authors:** Qiang Zhang, Haidong Xu, Dongming Zheng, Haonan Cheng, Zhenrui Liu, Chen Yang

**Affiliations:** 1 Textile Inspection and Testing Institute, Jiangxi Provincial Institute of Inspection and Testing, Nanchang 330052, China; 15180160317@163.com (Q.Z.); 13970096535@139.com (D.Z.); 13007210408@126.com (Z.L.); 2 College of Textile and Fashion, Hunan Institute of Engineering, Xiangtan 411104, China; x8340706461@163.com; 3 School of Fashion Engineering, Jiangxi Institute of Fashion Technology, No. 108, Lihu Middle Avenue, Xiangtang Economic Development Zone, Nanchang 330201, China; 4 Wuhan Textile University Gongqingcheng Textile and Garment Industry Research Institute, 2nd Floor, Down Museum, Yaya Industrial Park, Gongqingcheng Industrial New Area, Jiujiang 332020, China

**Keywords:** PET microplastics, textile microfibers, microfiber release, ICP-AES, FTIR

## Abstract

Textile-derived microplastics (MPs), particularly polyethylene terephthalate (PET) microfibers from infant garments, are an emerging concern due to potential early-life exposure and the vulnerability of infant physiological systems. However, existing analytical methods are often limited by complexity or poor suitability for larger, irregular textile fibers, with techniques such as SP-ICP-MS being less effective in this size domain. In this study, household laundering conditions were simulated (20–40 °C) to investigate PET microfiber release from infant outerwear. A combined ICP-AES and FTIR workflow was developed for quantitative and qualitative analysis. Washing was simulated using a color fastness tester, and samples were pretreated via Fenton oxidation and NaBr density separation. Carbon measured by ICP-AES was converted to PET mass, while FTIR confirmed polymer identity. The results showed that PET microfiber release increased from 1.75 to 2.53 mg with rising temperature, averaging 2.18 mg. FTIR peaks at 1713, 1251, and 1090 cm^−1^ confirmed PET as the dominant polymer. This study developed a detection method integrating ICP-AES quantification and FTIR qualitative characterization for MPs. In contrast to the SP-ICP-MS approach limited by fiber morphology and size, the established method realizes the qualitative and quantitative analysis of MPs to some extent and offers supporting data for the contamination control of MPs in infant apparel.

## 1. Introduction

Microplastics (MPs)—generally defined as solid plastic particles smaller than 5 mm in size—consist primarily of high-molecular-weight polymers blended with functional additives like antioxidants and heat stabilizers [1,2,3,4,5]. What makes these particles a persistent environmental and health concern is their inherent durability, high surface reactivity, and ability to adsorb co-occurring contaminants, all properties that let them interact readily with living organisms and diverse environmental matrices [6,7,8]. For this reason, pinning down the primary sources and release patterns of MPs is a critical foundation for accurate exposure assessment and the design of targeted source-control strategies.

To date, most MP detection research has focused on standard environmental matrices: seawater, freshwater, wastewater, and sediments, among others. Yet, textile products represent one of the most significant—and arguably most actionable—sources of fibrous MPs. Synthetic garments shed microfibers across their entire life cycle, from daily wear and mechanical abrasion to regular laundering and tumble drying [9,10]. As a representative fiber, PET is widely used at low cost with frequent human exposure. Its stable chemical properties and environmental recalcitrance bring greater potential health risks to infants. PET fabrics, in particular, are prone to pilling and surface fuzzing; during washing cycles, the combined force of hydrodynamic agitation and fabric-on-fabric friction speeds up fiber damage and eventual breakage. Higher wash and dry temperatures can weaken fiber structure even further, driving polymer swelling, loosening internal molecular bonds, and cutting tensile strength—all factors that raise the odds of microfiber shedding.

Prior work has shown that synthetic garments release measurable amounts of fibers in a single wash, and total shed mass varies widely depending on fabric type, yarn structure, wash load size, and specific operating conditions [9,10]. Zhao et al. [11], for example, found that microfiber release from denim fabrics climbed alongside wash load, while water temperature had a more nonlinear effect on overall shedding behavior.

Infants are an especially vulnerable group here, as their skin barrier, respiratory defense systems, and gastrointestinal function are all still in early development [12,13]. Their near-constant close contact with textiles opens up multiple potential exposure pathways: direct dermal contact, inhalation of airborne released fibers, and even incidental ingestion via hand-to-mouth behavior [14,15,16,17]. A growing body of experimental and review evidence indicates that inhaled or ingested MPs can interact with respiratory and gastrointestinal epithelial barriers, trigger local inflammatory responses, and compromise tissue integrity [18,19]. Wu et al. [20] expanded on this line of work, documenting both cytotoxic effects and transcriptomic changes in human Caco-2 intestinal cells after exposure to polystyrene microbeads. Taken together, these findings make clear that reliable, practical methods for characterizing MP release from laundered textiles are needed to inform evidence-based strategies for reducing exposure.

Current standard methods for MP identification include microscopy paired with FTIR imaging [21,22], Raman micro-imaging [23], and pyrolysis–gas chromatography–mass spectrometry (Py-GC-MS) [24,25]. While all of these deliver high-value chemical compositional data, they come with notable practical drawbacks: labor-intensive pretreatment steps, long analysis run times, or reliance on specialized data processing workflows.

ICP-based techniques offer a promising alternative, enabling mass- or element-based quantification of MP samples. Bolea-Fernandez et al. [26] previously demonstrated that SP-ICP-MS can detect MPs by monitoring carbon content, using polystyrene microspheres as a reference material. That work, however, was built around uniform, spherical model particles, and the method does not perform well when applied to large, irregularly shaped textile microfibers. ICP-AES, by contrast, works well for bulk elemental quantification as long as samples are properly digested and calibrated, though it cannot confirm particle morphology or polymer identity on its own, and it requires pairing with a complementary technique like FTIR for full characterization.

Against this backdrop, this study simulates PET microfiber release from infant outerwear under controlled laboratory washing conditions, and develops a combined analytical workflow that integrates ICP-AES with FTIR spectroscopy. ICP-AES is used to estimate total PET microfiber mass based on carbon concentrations measured in the digested sample suspension, while FTIR provides chemical confirmation of the PET polymer and supports qualitative identification of the collected fibers. It is worth noting that because ICP-AES measures bulk elemental content rather than individual particle-level properties, it cannot directly produce data on particle size distribution or morphological features; these details must be interpreted alongside complementary microscopic or spectroscopic evidence.

## 2. Results

### 2.1. Nebulization and Atomization of Microplastic Suspensions in ICP-AES

When using a standard quartz concentric nebulizer paired with a quartz cyclonic spray chamber, transport efficiency for dissolved analytes can exceed 2%. For suspended microplastic particles, by contrast, this efficiency may decrease to below 0.1%, and larger particles carry a notable risk of system clogging. To boost nebulization performance and widen the workable particle size range, a Ryton Scott dual-channel spray chamber was adopted for this study.

This spray chamber is conventionally matched with a gem-tip cross-flow nebulizer, a setup known to reduce clogging when feeding large, rigid MP particles into the instrument. Its dual makeup-gas system creates tangential gas flow along the inner chamber walls, which cuts down on particle loss from wall deposition. A practical benefit of this design is that the makeup-gas flow can be tuned independently of the nebulizer gas flow, allowing control over particle residence time in the plasma—and in turn, shaping volatilization, atomization, and carbon emission behavior.

### 2.2. Quantitative Detection by ICP-AES

Quantification by ICP-AES is rooted in atomic emission spectroscopy: atoms and ions excited in the plasma emit radiation at characteristic wavelengths. Under suitable experimental conditions, emission intensity is approximately linear within the working concentration range. Under these conditions, a series of standard solutions with known concentrations are prepared. A calibration curve of emission intensity versus concentration is established via least-squares linear regression, enabling the quantitative determination of elemental content in unknown samples.

**Figure 1:**
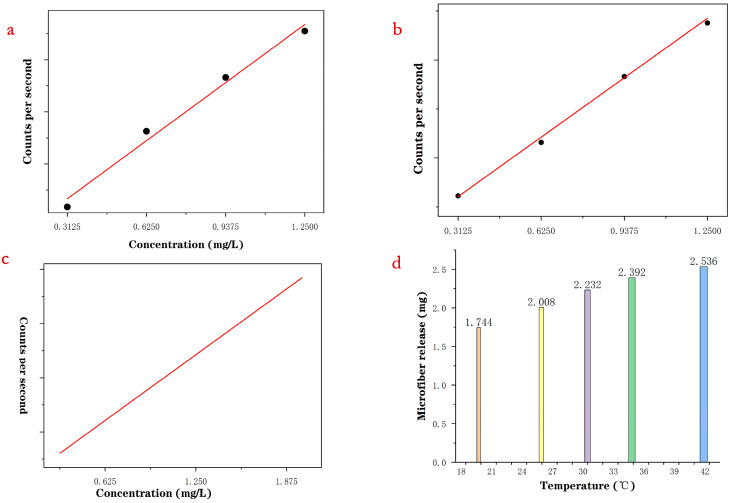
(**a**) Ordinary least squares (OLS)-fitted standard calibration curve; (**b**) standard calibration curve generated via weighted least squares (WLS); (**c**) plot of predicted concentrations; (**d**) bar chart showing microplastic release from PET at various temperature gradients.

As shown in Figure 1a, the OLS calibration curve displayed heteroscedasticity: fitting errors were small at higher concentrations, whereas relative errors were larger at lower concentrations. This discrepancy indicates unequal error variance across the calibration range.

Because OLS assumes equal error variance for all data points, the regression may be dominated by high-concentration points and may produce larger errors for low-concentration samples. Weighted least squares (WLS) was therefore used to improve calibration performance in the low-concentration range. The WLS objective function is expressed as follows:

$$w_{i}=\frac{1}{X_{i}^{2}}$$

where *X* denotes the concentration, and *w_i_* denotes the weight assigned to each calibration point.

The $\frac{1}{X_{i}^{2}}$ weight is adopted to account for concentration-dependent signal variance, reducing the influence of high-concentration points and improving low-concentration fitting performance. The fitting results are shown in Figure 1b. By assigning weights according to error level, WLS reduced the influence of heteroscedasticity and improved the fit in the low-concentration region. This approach decreased quantitative error for trace samples and produced results that were more consistent with the experimental data.

We plugged the measured intensity values into the WLS calibration curve to derive predicted concentrations, as displayed in Figure 1c. These predicted elemental concentrations were then converted to PET microfiber mass using the external-standard calculation, with the full quantitative results summarized in Table 1.

Figure 1d shows a bar chart of microplastic (MP) release data plotted based on three tests per sample group and three sample sets at each temperature. The bar chart indicates that microplastic release increases continuously with rising temperature, but the rate of increase slows slightly at the two temperature points of 35 °C and 40 °C. The average MP release was approximately 2.18 mg, with no obvious outliers, indicating good experimental reproducibility. One-way analysis of variance (ANOVA) was performed to compare microfiber release amounts at different washing temperatures. A highly statistically significant intergroup difference was detected (F = 56.6, *p* < 0.001). Pearson correlation analysis revealed a significant positive correlation between washing temperature and microfiber release (r = 0.975, *p* < 0.001). These results suggest washing temperature may influence PET microfiber shedding. That said, the small sample size and limited temperature range prevent a robust conclusion about linearity, particularly for temperatures above 40 °C. Further experiments with larger sample sizes and a broader temperature range will be required to explore this relationship more fully.

### 2.3. Qualitative Analysis by Fourier Transform Infrared Spectroscopy (FTIR)

Since ICP-AES only picks up elemental signals, it cannot confirm polymer type on its own. We therefore used FTIR to verify the chemical composition of the retained microfibers. During FTIR analysis, molecules absorb infrared radiation at specific frequencies and undergo vibrational transitions, yielding characteristic absorption bands.

**Figure 2:**
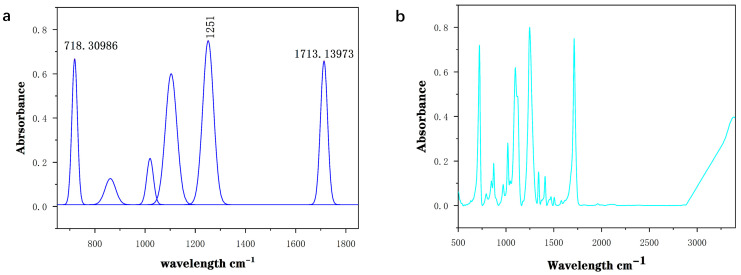
(**a**) Peak deconvolution results fitted with Origin; (**b**) original FTIR spectrum.

FTIR instruments collect interferometric data across a broad wavelength range. The raw time-domain interferogram is converted to a frequency-domain spectrum using a fast Fourier transform (FFT), making it possible to identify both the chemical composition and molecular structure of a sample. In this work, we used FTIR to analyze the MPs captured on 0.45 μm filter membranes; the infrared spectra of microfibers shed from the PET fabrics are shown in Figure 2.

The broad absorption band spanning 3000–3400 cm^−1^ is attributed to hydroxyl (–OH) stretching vibrations, most likely from residual moisture or hydrogen-bonded species present in the sample. The peak centered at approximately 3388.32 cm^−1^ specifically corresponds to hydrogen-bond-associated vibrations. The sharp peak at 1713 cm^−1^ arises from carbonyl (C=O) stretching, while the prominent bands near 1251 cm^−1^ and 1090 cm^−1^ originate from ester C–O stretching [27,28]. The band at 1161 cm^−1^ comes from asymmetric C–O–C stretching within the PET molecular backbone, and the smaller peaks around 1003 cm^−1^ and 986 cm^−1^ match C–O stretching and aromatic ring skeleton vibrations, respectively. All together, these signature bands confirm that the retained microfibers are PET.

## 3. Discussion

In this work, we developed a combined qualitative and quantitative workflow for analyzing relatively large, textile-derived PET microplastics by pairing ICP-AES with FTIR spectroscopy. Using a washing fastness tester to simulate routine laundering, we examined PET microfiber release from infant outerwear across a 20–40 °C temperature gradient.

Our ICP-AES approach estimates total PET mass indirectly by measuring carbon concentration in the digested sample suspension, which lets us sidestep the particle-size limitations inherent to particle-resolved SP-ICP-MS. For its part, FTIR analysis confirmed polymer identity via the characteristic absorption bands of PET, verifying that the collected fibers were textile-derived PET microfibers.

This study has notable limitations. We only assessed temperature as an influencing variable, within a narrow 20–40 °C range, and the total number of test specimens was limited. Future work should expand the tested temperature range and systematically evaluate other key factors: washing duration, detergent formulation, wash load size, fabric structure, garment service age, and drying conditions, among others. Blank correction protocols, recovery tests, and matrix effect evaluations also need further refinement to improve the reliability of carbon-based ICP-AES quantification. Broader testing across infant garments of different material compositions would also build a stronger evidence base for setting microfiber release limits and developing targeted mitigation strategies for textile microplastics.

## 4. Materials and Methods

### 4.1. Instruments and Reagents

Hydrogen peroxide (H_2_O_2_, 30% *v*/*v*, guaranteed reagent grade, Damao Chemical Reagent Factory, Tianjin, China) and sodium bromide (NaBr, guaranteed reagent grade, Damao Chemical Reagent Factory, Tianjin, China) were obtained from Damao Chemical Reagent Factory. Ferrous sulfate (FeSO_4_, analytical reagent grade, Zhonglian Chemical Reagent Co., Ltd., Tianjin, China) and sodium hydroxide (NaOH, analytical reagent grade, Zhonglian Chemical Reagent Co., Ltd., Tianjin, China) were purchased from Zhonglian Chemical Reagent Co., Ltd. (Tianjin, China). PET microspheres (Analytical Reagent, Macklin, Shanghai, China) with a nominal particle size of 50 μm were employed as the reference material for method validation. To cut down background plastic contamination, all experimental operations were performed exclusively with glass stirring rods and standard glassware. Grade 1 ultrapure water compliant with the GB/T 6682 standard [29] was used for every step of solution preparation and sample processing. An outdoor jacket (100% polyester, woven structure; supplied by Qingdao Yilubeier Garment Co., Ltd., Qingdao, China) was used in this experiment.

A washing color fastness tester (Quanzhou Meibang Instrument Co., Ltd., Quanzhou, China) was adopted to simulate controlled household laundering conditions. Carbon content quantification was run on an inductively coupled plasma atomic emission spectrometer (ICP-AES; PerkinElmer Inc., Shanghai, China), and its detailed operating parameters are listed in Table 2. A Nicolet iS20 Fourier transform infrared (FTIR) spectrometer (Thermo Fisher Scientific, Waltham, MA, USA) was used to verify the chemical composition of collected microfibers. All weighing procedures were carried out on an analytical balance. For filtration steps, glass fiber filter membranes (0.45 μm pore size, 50 mm diameter, Shanghai Xingya Purification Materials Factory, Shanghai, China) were selected for sample separation. Peak deconvolution and curve fitting were performed using OriginPro 2021 software (OriginLab Corporation, Northampton, MA, USA).

### 4.2. Sample Preparation

First, PET outerwear samples were placed in a constant temperature–humidity chamber for 24 h, maintained at 20.8 °C and 66.7% relative humidity. This conditioning step standardizes fabric moisture regain, which helps reduce fluctuations in sample mass and microfiber release that stem from variable moisture content.

**Figure 3:**
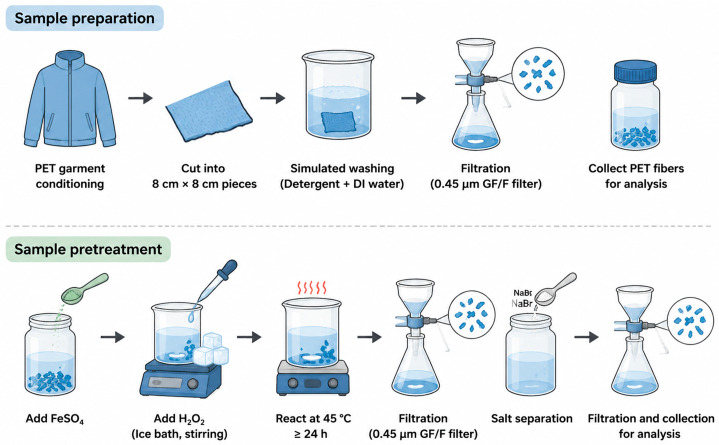
Experimental workflow of microplastic treatment for washed PET jackets: sample preparation, density separation, oxidative digestion.

Once conditioned, the fabric was cut into five sets of 8 cm × 8 cm test specimens. For each washing trial, 0.96 g of laundry powder was dissolved in 160 mL of ultrapure water, and specimens were washed in the color fastness tester to mimic routine household laundering. Detailed washing parameters are listed in Table 3. The overall workflow for sample preparation, density separation, and oxidative digestion is illustrated in Figure 3.

After each wash cycle, the resulting wastewater was collected and filtered through a 0.45 μm glass fiber membrane to trap released PET microfibers. We rinsed the inner walls of the container and the membrane surface repeatedly with ultrapure water, then quantitatively transferred all retained microfibers into clean glass sample vials.

### 4.3. Sample Pretreatment

A total of 0.25 g of FeSO_4_ was weighed and added to the sample, which was then diluted to a final volume of 100 mL with ultrapure water. To prevent the exothermic reaction induced by hydrogen peroxide addition from damaging the PET structure, the sample vial was placed in an ice-cooled thermostatic water bath. A total of 100 mL of 30% (*v*/*v*) H_2_O_2_ was slowly added dropwise under continuous stirring. The pH was adjusted to 3.0 with NaOH during the reaction. After the vigorous reaction subsided, the mixture was transferred to a thermostatic water bath at 45 °C and incubated for at least 24 h until the supernatant became completely clear.

Subsequently, the mixture was vacuum-filtered through a 0.45 μm glass fiber filter, and the retentates on the filter membrane were collected. The retentates were thoroughly rinsed into a clean glass vial with ultrapure water, diluted to a final volume of 50 mL, and placed in a thermostatic water bath at 25 °C. Excess NaBr was added to the sample, followed by stirring until undissolved crystals precipitated at the bottom. The mixture was allowed to stand at constant temperature for phase separation until the supernatant was clarified. The supernatant was then filtered through a 0.45 μm glass fiber filter. The retentates on the filter were rinsed repeatedly with ultrapure water and collected in a clean container for subsequent analysis.

### 4.4. PET Standards

PET microparticle suspensions were prepared from commercially available particles. Standard particles of 0.5 mg, 1 mg, 1.5 mg, and 2 mg were weighed individually, diluted with ultrapure water, and brought to a final volume of 1 L. The stock suspensions were then sonicated for 1 min using a dual-frequency digital ultrasonic cleaner operated at 45 kHz. No surfactant was added for suspension stabilization, as organic surfactants would increase the dissolved organic carbon (DOC) content and consequently elevate the detection limit of particle size determination.

### 4.5. Method Validation

Procedural blanks were implemented for blank correction throughout the testing process, and the corresponding blank signal intensity was subtracted from the raw results of subsequent ICP-AES measurements. The pretreatment protocol for PET fibers was adapted from the methods reported by Löder et al. [30] and Pfohl et al. [31,32], which had a verified recovery rate of over 90% for PET fibers, as documented in the original studies. In addition, a blank control group consisting solely of water and detergent was set under conditions identical to the experimental group. After the same pretreatment, the carbon signal difference between the blank and ultrapure water was approximately 2%, which exerted no significant impact on the final quantification.

Method repeatability was evaluated by performing triplicate parallel measurements on the same sample, yielding a relative standard deviation (RSD) of 6.816% (RSD < 20%). The quantitative calculation of PET was based on the following equation, where (*Wc*) denotes the mass fraction of carbon element, (*Pc*) denotes the measured net mass concentration of carbon, and *V* denotes the total volume of the test sample solution:

$$Mpet=\frac{Pc\times V}{Wc}$$

Additionally, three blank samples spiked with 1 mg of PET standard were analyzed to determine the analytical limit of detection (LOD) and limit of quantitation (LOQ). The LOD and LOQ were calculated as 3-fold and 10-fold the standard deviation of the measurement results, respectively. The matrix spike recovery test was performed by spiking PET standard into the sample matrix, and an average spike recovery of 94.3% was determined.

## Figures and Tables

**Table 1:** Quantitative Results of PET Microfibers in Washing Wastewater.

Sample	Concentration (mg/L)	Washing Temperature (°C)	Fiber Mass (mg)
1	2.18	20	1.744
2	2.20	20	1.760
3	2.22	20	1.776
4	2.51	25	2.008
5	2.55	25	2.040
6	2.54	25	2.032
7	2.79	30	2.232
8	2.72	30	2.176
9	2.77	30	2.216
10	2.99	35	2.392
11	2.89	35	2.312
12	2.92	35	2.336
13	3.17	40	2.536
14	3.15	40	2.520
15	3.17	40	2.536

**Table 2:** Operating Parameters of the ICP-AES Instrument.

Parameter	Value
RF Power	1400 W
Argon Gas Flow Rates	
Plasma Gas	12 L min^−1^
Auxiliary Gas	0.5 L min^−1^
Nebulizer Gas	0.7 L min^−1^
Makeup Gas	0.2 L min^−1^
Sample Uptake Rate	1.5 mL min^−1^
Observation Height	15 mm
Total Acquisition Time	50 s

**Table 3:** Experimental Design of Washing Tests.

Sample	Mass (g)	Washing Temperature (°C)	Washing Time (min)	Mass-Specific Microfiber Release (mg/g)
1	4.02	20	40	0.43383
2	4.09	20	40	0.43032
3	4.11	20	40	0.43212
4	4.06	25	40	0.49458
5	4.16	25	40	0.49038
6	4.09	25	40	0.49682
7	4.12	30	40	0.54175
8	4.21	30	40	0.51686
9	4.16	30	40	0.53269
10	4.32	35	40	0.5537
11	4.27	35	40	0.54145
12	4.07	35	40	0.57396
13	4.12	40	40	0.61553
14	4.21	40	40	0.59857
15	4.18	40	40	0.6067

## Data Availability

The data presented in this study are available on request from the corresponding author.
